# Hemodialysis as long term treatment: Patients satisfaction and its impact on quality of life

**DOI:** 10.12669/pjms.37.2.2747

**Published:** 2021

**Authors:** Muhammad Sajid Iqbal, Quratulain Iqbal, Shahreen Iqbal, Sania Ashraf

**Affiliations:** 1 Dr. Muhammad Sajid Iqbal, MBBS, FCPS (Medicine). Senior Registrar, (Med) Lahore General Hospital, Lahore, Pakistan; 2 Dr. Quratulain Iqbal, MBBS. Research Volunteer, Lahore General Hospital, Lahore, Pakistan; 3 Dr. Shahreen Iqbal, MBBS. Research Volunteer, Lahore General Hospital, Lahore, Pakistan; 4 Dr. Sania Ashraf, MBBS. Post Graduate Resident (Nephrology), Lahore General Hospital, Lahore, Pakistan

**Keywords:** Satisfaction, Hemodialysis, ESRD, Quality of life

## Abstract

**Objective:**

To determine the level of satisfaction as hemodialysis a long term treatment and quality of life in patients off End Stage Kidney Disease ESKD on hemodialysis.

**Methods:**

A cross-sectional study was carried out from January to April 2019 in hemodialysis unit of Lahore General Hospital on 141 ESKD patients by using self-designed questionnaire after informed consent.

**Results:**

Majority (82.56%) of the participants were satisfied with the care provided at the dialysis center. except with the time spent with doctor and 36.9% were not satisfied with their cannulation technique for dialysis. About 89.9% were satisfied with the knowledge provided to them about self-care. Satisfaction is subjective well-being in different aspects of life, including mental health and behavior of people experiencing serious health concerns. Quality of Life (QOL) is defined as “perception of one’s position in life, in the light of his culture and customs, consisting someone’s goals, standards or expectations. Financial problems to the patient was limited to the transportation as dialysis session and erythropoietin were free, but 54.1% of the patients were unable to earn due to their disease even those who were working ,80% of them had to take the day off for dialysis. The financial burden and debilitating illness didn’t cause separation/divorce from spouse but led to increased frequency of scuffles. Among the unmarried population, 40% of it does not want to start a relationship and 40% is facing difficulties in finding a partners while 97.9% of the population is satisfied with the psychological and emotional support of family.

**Conclusion:**

Most patients were satisfied with their decision of opting hemodialysis as treatment and care provided at dialysis centre, although Quality of Life was badly affected in terms of financial and psycho-social aspects. Employed, married with good income have good quality of life. Loopholes of unit environment and health education were also exposed. Despite the medical advancement and emerging techniques to make dialysis better, the outcome of hemodialysis has yet to reach a safe level and more work should be done to improve patient’s outcome.

## INTRODUCTION

Chronic Kidney Disease(CKD) is one of the leading causes of death worldwide. Chronic kidney disease was the cause of 956,000 deaths globally in 2013, up from 409,000 deaths in 1990.^1^ All individuals with a glomerular filtration rate (GFR) <60 ml/min/1.73 m^2^ for three months are classified as having chronic kidney disease, irrespective of the presence or absence of kidney damage. The condition of individuals with CKD who require renal replacement therapy is referred to as the end-stage kidney disease (ESRD). Hemodialysis is an alternate of renal functioning for survival, either temporary (waiting for renal transplantation) or lifelong.^2^ It has many implications which affect physical, psychological or social aspect of life e.g. fatigue, bone pain, dyspnea, low self-esteem, anxiety, depression etc.^3^

Advent of RRT has significantly increased life expectancy in ESRD patients, but these are not truly curative rather life-extending treatments. While renal replacement therapies can maintain and prolong life, but the quality of life is severely affected, not only via disease but also psychosocial factors.^4^ Most of the studies and trials, so far, have largely focused on biomarker endpoints and quantitative outcome to evaluate care but level of satisfaction in dialysis patients is highly dependent on normalization of their lives and how regular dialysis affect them financially and socially.^5^ As patients on hemodialysis spend significant amount of time in dialysis center, the satisfaction with care provided there has an important impact on quality of their lives and it improves patient-outcome.^6^,^7^ Better communication of staff with patients, plays important role for better results.^8^ Care provided at hospital is not only limited by doctors but nurses, paramedical staff, technician and managers all play a vital role. Moreover, Education level, earning and family support, age, marital status all affect patients adherence to treatment and satisfaction.^9^

Assessment of patient satisfaction is becoming necessary to evaluate the healthcare outcomes, as twice yearly carried out in USA.^10^ The objective of our study was to describe patient’s satisfaction with care provided at dialysis center and impact of dialysis on different aspects of their lives such as financial, social, marital and personal affairs.

## METHODS

A cross sectional study among 141 patients who were undergoing hemodialysis for more than three months, twice weekly in fixed shifts, at dialysis center of Lahore General Hospital (Pakistan), coming from Lahore and its near periphery. Study was conducted from January to April 2019 after ethical committee approval (AMC/PGMI/LGH/Article Research No/0058-18, Dated 20, May 2018) and Informed consent taken from all patients. Patients on Hemodialysis less than three months, acute renal failure, having dementia or cognitive impairment were not included in study.

Demographic Data included age, gender, education status, material status, residence, employment status and duration of hemodialysis was collected. Satisfaction and quality of life was assessed by using the World Health Organization Quality of life (WHOQOL-BREF) questionnaire, simplified into three categories; 1^st^ satisfaction with health care provider & services along with education provided for self-care, 2^nd^ satisfaction with economical expenses for health care and their income, 3^rd^ satisfaction regarding personal and social relationships. Survey was conducted with help of facilitators, who explains questions to those who were unable to read or understand.

Data was analyzed according to objectives by using SPSS version 22; a descriptive statistical analysis was undertaken. Continuous were expressed as mean ± SD, whereas categorical variables were expressed as frequency. One-way analysis of variance (*F*-Test) was used to test the statistical difference of mean age and income, T-test for comparison and chi-square test for any association between categorical variables was used. Statistical significant p-value considered if less than 0.05.

## RESULTS

A total of 141 patients undergoing regular hemodialysis at dialysis center of Lahore General Hospital were included in the survey; out of which 98 (69.5%) were males and 43 (30.5%) were females. 89.4% of them were married and 10.6% were not. 24% of our participants had formal education and 75.8% were illiterate. The mean duration of dialysis in our study population was three to four years; of these 12% have been on hemodialysis for more than five years, 31.9% for 3-5 years, 31.2% for 1-3 years and 24.8% for less than a year.

**Fig.1:**
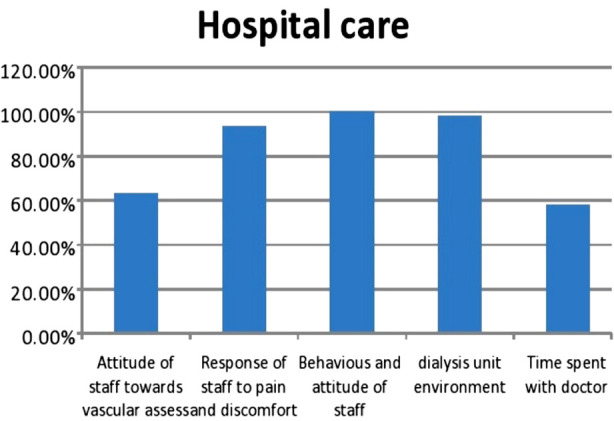
Satisfaction parameters in study population regarding Hospital Care.

Majority of the participants were found to be satisfied with the care provided at the dialysis center; except the time spent with doctor (58.2%), which makes it least satisfactory variable. While 36.9% of study population was not satisfied with the approach of staff towards their vascular access; major concern being cannulated by newer staff nurses who were not trained well-enough. Most of the patients were satisfied with other aspects of hospital care, as shown in Fig.1.

Majority (89.4%) were satisfied with the knowledge provided to them about self-care, whereas about 19.1% of cohort was not satisfied with the knowledge provided to them about hemodialysis and its possible complications.

**Fig.2:**
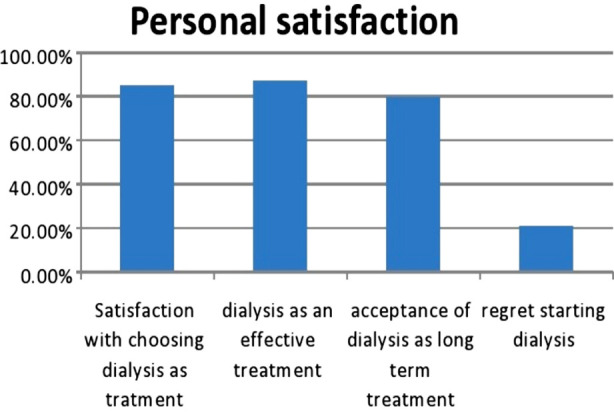
Patients Satisfaction Status about Hemodialysis.

### Quality of life

Total 69.5% (98 patients) were employed (male 87.7%, female 12.3%). 6.3% were government employees, 36.1% private employees, 26.95% self-employed, house wife were 21.9%, retired (8.5%) while remaining 43(30.5%) were jobless. Majority (61.7%) were low income (earning less than 10,000 per month). Transportation cost was less than 500 rupees for 73.5% of our studied population.

However, 54.1% of employed patients were unable to earn due to their disease, decreasing the net income of the family and leading to financial troubles. Even among those who were able to work despite ESRD, 80% had to take the day off on the day of dialysis.

### Social

The financial burden and debilitating illness did not cause separation/divorce from spouse for any of our participants; but led to more frequent scuffles between them. In our unmarried population it has led to difficulty in starting new relationships; 40% of them don’t want to start any new relationship, 40% were facing difficulties while searching for life partners and dialysis dependence lead to broken engagement for 20% them. Similarly, 15.6% of patients were having difficulty in their relationship with their friends. Overall, 97.9% of population is satisfied with the psychological and emotional support provided to them by their families.

Regarding personal satisfaction, 85-87% was satisfied in choosing dialysis and its effectiveness, 20.6% of the cohort has not yet accepted hemodialysis as a long term treatment leading to the regret that they should not have started dialysis in the first place, because lack of prognostic awareness.

## DISCUSSION

The condition of individuals with Chronic kidney Disease, who require either of the two types of renal replacement therapy (dialysis or transplant), is referred to as the end-stage kidney disease. The prevalence of ESRD seem to double every 10 years.^11^

Hemodialysis is the most frequently used treatment for ESRD. It has massive impact on lives of patient as they have to visit hospital twice or thrice per week, even more than that in some cases, forcing them to spend significant amount of their time in the hospital so the care provided there, the psychological support from the health-care providers plays significant role in improving the quality of their lives.^12^

Our most of patients were illiterate 75.8% (may read and write), 16.3% were educated from primary to matric and 7.8% above matric, comparing to Al-Abri R et al 13 48.1% can read and write and 26.6% having primary schooling.

Patient’s satisfaction to provided hospital care is 82.56%, which is higher than many other studies across world i.e 41%, 50% and 47% observed by Park, Bayoumi M (Egypt) and Sharma M respectively^14^-^16^ but almost equal to AL-Jumaih A in Saudi Arabia (81.5%).^17^ However 36.9% of patients were not satisfied with healthcare staff especially vascular access and cannulation by untrained or younger staff nurse which is higher than Ndambuki J and Door (Sudan) 23.8% & 11.4% respectively.^18^,^2^ Satisfaction regarding time spent with doctors is 58% which is comparable 64.6% by Magda Bayoumi.^15^

Majority of our study participants (90-100%) accepted of the services provided by health care professionals like response of nursing staff to patient’s pain and discomfort during the process of hemodialysis, attitude and commitment of doctors in dealing with emergency situations like fits, hypo/hypertension, and arrhythmias during hemodialysis.

About 54.1% of employed patients showed their financial concerns due to their inability to earn because of their illness. About 80% patients had to take off from their work on the day of dialysis which poses economic burden on them especially on those with working on daily wages. Though dialysis provided in the studied facility was free of cost with little or almost no expenditure on additional medication (like erythropoietin, iron supplementation or antibiotics if needed) and nutritional supplements used during dialysis, but still large number of patients mentioned the importance of appropriate income in their lives and the various troubles they have to face due to their financial constraints. As majority of our patients were low incomes (61.7%) similar observation noted by Anees et al.^19^ Thus financial support of these patients and their families by the government and social welfare organizations may play an important role in improving the quality of life of these patients.

Regarding personal satisfaction level in choosing hemodialysis as a life-long treatment modality, it was found that patients mostly rely on their health care professionals/doctors in decision of dialysis modality. Lack of adequate discussions and guidance affect patient’s well-being both in the form of social and financial aspects.^20^,^21^ Almost 85.1% of our studied cohort revealed their satisfaction in choosing hemodialysis as a life-long treatment option with 87.1% revealing it as an effective mode of treatment. But 20.6% of these patients showed their regret in considering hemodialysis as treatment option to their ailment, similar observation noted by Saeed F.^22^

Stress is generally more in patients with any chronic illness than general healthy individuals which also affect their relationships with family members, spouse and friends.^23^ While all of our married patients revealed contentment in their social and personal relationships such as with their spouses and family members, which had positive impact on their life and health quality. However, 40% of the unmarried studied population revealed facing problems in establishing new relationships which led to increased anxiety and depression among these patients thereby hindering their quality of lives.

### Limitation to the study

Lack of anonymity lead to decreased response rate from the patients thereby biasing the results. Also data was collected over a short duration of time, if it had been collected over years a subtler response could have been achieved.

## CONCLUSION AND RECOMANDATIONS

Most patients were satisfied with their decision of opting hemodialysis as treatment and care provided at dialysis centre, although Quality of Life was badly affected in terms of financial and psycho-social aspects. Employed, married with good income have good quality of life. Loopholes of unit environment and health education were also exposed. Despite the medical advancement and emerging techniques to make dialysis better, the outcome of hemodialysis has yet to reach a safe level and more work should be done to improve patient’s outcome.

### Authors’ Contribution:

**MSI** conceived, designed and did statistical analysis and editing of manuscript.

**QI & SI** did data collection.

**SA** did data analysis, manuscript writing.

**MSI** takes responsibility and accountable all aspects of work in ensuring that questions regarding accuracy and integrity of work is appropriately investigated and resolved.

## References

[1] GBD 2013. Mortality and Causes of Death. Collaborators (17 December 2014). Global regional, and national age-sex specific all-cause and cause-specific mortality for 240 causes of death 1990–2013:A systematic analysis for the Global Burden of Disease Study 2013 (2015). Lancet.

[2] Door ZH, Mukhtar HF (2019). The Satisfaction of Patients on Maintenance Hemodialysis Concerning the Provided Nursing Care in Hemodialysis Units. IOSR J Nurs Health Sci.

[3] Stavropoulou A, Grammatikopoulou MG, Rovithis M, Kyriakidi K, Pylarinou A, Markaki AG (2017). Through the Patients Eyes:The Experience of End-Stage Renal Disease Patients Concerning the Provided Nursing Care. Healthcare [Internet].

[4] Kim K, Kang GW, Woo J (2018). The quality of life of hemodialysis patients is affected not only by medical but also psychosocial factors:A canonical correlation study. Korean Med Sci.

[5] Tong A, Sainsbury P, Carter SM, Hall B, Harris DC, Walker RG (2008). Patients'priorities for health research:focus group study of patients with chronic kidney disease. Nephrol Dial Transplant.

[6] Richardson MM, Paine SS, Grobert ME, Stidley CA, Gabbay E, Harford AM (2015). Satisfaction with Care of Patients on hemodialysis. Clin J Am Soc Nephrol.

[7] van der Veer SN, Arah OA, Visserman E, de Keizer NF, Abu-Hanna A, Heuveling LM (2012). Exploring the relationships between patient characteristics and their dialysis care experience. Nephrol Dial Transplant.

[8] Newell S, Jordan Z (2015). The patient experience of patient-centered communication withnurses in the hospital setting:a qualitative systematic review protocol. JBI Database Syst Rev Implement Reports.

[9] Batool Z, Nafees M, Ashraf R, Hayyat U, Anwar S (2018). Social Life of End Stage Renal Disease Patients. Pak J Med Biol Sci.

[10] Dad T, Tighiouart H, Lacson E, Meyer KB, Weiner DE, Richardson MM (2018). Hemodialysis patient characteristics associated with better experience as measured by the In-center Hemodialysis Consumer Assessment of Healthcare Providers and Systems (ICH CAHPS) survey. BMC Nephrol.

[11] Hill NR, Fatoba ST, Oke JL, Hirst JA, O'Callaghan CA, Lasserson DS (2016). Global prevalence of chronic kidney disease –A systematic review and meta-analysis. PLoS One.

[12] Bowling CB, Vandenberg AE, Phillips LS, McClellan WM, Johnson TM, Echt KV (2017). Older patients'perspectives on managing complexity in CKD self-management. Clin J Am Soc Nephrol.

[13] Al-Abri R, Al-Balushi A (2014). Patient satisfaction survey as a tool towards quality improvement. Oman Med J.

[14] Park GY, Yoo EK (2016). A study on quality of life in hemodialysis patients. Information (Japan).

[15] Bayoumi M, Guindy HA El, Ahmed A (2016). Patients ‟Satisfaction“with Care at Dialysis Unit. Int J Nurs Sci.

[16] Sharma M (2018). Satisfaction with care in hemodialysis unit among Maintenance Hemodialysis (Mhd) patients. Int J Dev Res.

[17] AL-Jumaih A, Al-Onazi K, Binsalih S, Hejaili F, Al-Sayyari A (2011). Study of Quality of Life and its determinants among hemodialysis patients using the KDQOL-SF instrument in one center in Saudi Arabia. Arab Nephrol Transplant.

[18] Ndambuki J (2013). The level of Patients Satisfaction and Perception on Quality of Nursing. Open J Nurs.

[19] Anees M, Batool S, Imtiaz M, Ibrahim M (2018). Socio-economic factors affecting quality of life of Hemodialysis patients and its effects on mortality. Pak J Med Sci.

[20] Van Biesen W, van der Veer SN, Murphey M, Loblova O, Davies S (2014). Patients'perceptions of information and education for renal replacement therapy:an independent survey by the European Kidney Patients'Federation on information and support on renal replacement therapy. PLoS One.

[21] Juergensen E, Wuerth D, Finkelstein SH, Juergensen PH, Bekui A (2006). Finkelstein FO:Hemodialysis and peritoneal dialysis:Patient's assessment of their satisfaction with therapy and the impact of the therapy on their lives. Clin J Am Soc Nephrol.

[22] Saeed F, Ladwig S, Sardar M, Duberstein PR (2019). Why do some patients regret their decision to initiate dialysis?(S865). J Pain Symptom Manag.

[23] Hickman RL, Douglas SL (2010). Impact of Chronic Critical Illness on the Psychological Outcomes of Family Members. AACN Adv Crit Care.

